# Prenatal ambient air pollution exposure and child weight trajectories from the 3rd trimester of pregnancy to 2 years of age: a cohort study

**DOI:** 10.1186/s12916-023-03050-y

**Published:** 2023-09-07

**Authors:** Nan Ji, Mark Johnson, Sandrah P. Eckel, William J. Gauderman, Thomas A. Chavez, Kiros Berhane, Dema Faham, Fred Lurmann, Nathan R. Pavlovic, Brendan H. Grubbs, Deborah Lerner, Rima Habre, Shohreh F. Farzan, Theresa M. Bastain, Carrie V. Breton

**Affiliations:** 1 Division of Environmental Health, Department of Population and Public Health Sciences, Keck School of Medicine, University of Southern California, 1845 N Soto St, MC 9239, Los Angeles, CA 90039 USA; 2 Heluna Health, Los Angeles, CA 91746 USA; 3 Department of Biostatistics, Mailman School of Public Health, Columbia University, New York, NY 10032 USA; 4 Sonoma Technology Inc., Petaluma, CA 94954 USA; 5 Department of Obstetrics and Gynecology, Keck School of Medicine, University of Southern California, Los Angeles, CA 90089 USA; 6 Eisner Health, Los Angeles, CA 90015 USA

**Keywords:** Prenatal air pollution exposure, In utero growth, Infant weight trajectory, Marginalized populations

## Abstract

**Background:**

Prenatal air pollution exposure may increase risk for childhood obesity. However, few studies have evaluated in utero growth measures and infant weight trajectories. This study will evaluate the associations of prenatal exposure to ambient air pollutants with weight trajectories from the 3rd trimester through age 2 years.

**Methods:**

We studied 490 pregnant women who were recruited from the Maternal and Development Risks from Environmental and Social Stressors (MADRES) cohort, which comprises a low-income, primarily Hispanic population in Los Angeles, California. Nitrogen dioxide (NO_2_), particulate matter < 10 µm (PM_10_), particulate matter < 2.5 µm (PM_2.5_), and ozone (O_3_) concentrations during pregnancy were estimated from regulatory air monitoring stations. Fetal weight was estimated from maternal ultrasound records. Infant/child weight measurements were extracted from medical records or measured during follow-up visits. Piecewise spline models were used to assess the effect of air pollutants on weight, overall growth, and growth during each period.

**Results:**

The mean (SD) prenatal exposure concentrations for NO_2_, PM_2.5_, PM_10_, and O_3_ were 16.4 (2.9) ppb, 12.0 (1.1) μg/m^3^, 28.5 (4.7) μg/m^3^, and 26.2 (2.9) ppb, respectively. Comparing an increase in prenatal average air pollutants from the 10th to the 90th percentile, the growth rate from the 3rd trimester to age 3 months was significantly increased (1.55% [95%CI 1.20%, 1.99%] for PM_2.5_ and 1.64% [95%CI 1.27%, 2.13%] for NO_2_), the growth rate from age 6 months to age 2 years was significantly decreased (0.90% [95%CI 0.82%, 1.00%] for NO_2_), and the attained weight at age 2 years was significantly lower (− 7.50% [95% CI − 13.57%, − 1.02%] for PM_10_ and − 7.00% [95% CI − 11.86%, − 1.88%] for NO_2_).

**Conclusions:**

Prenatal ambient air pollution was associated with variable changes in growth rate and attained weight from the 3rd trimester to age 2 years. These results suggest continued public health benefits of reducing ambient air pollution levels, particularly in marginalized populations.

**Supplementary Information:**

The online version contains supplementary material available at 10.1186/s12916-023-03050-y.

## Background

The steady rise in childhood obesity coupled with its later life consequences presents a major public health challenge that urgently needs effective prevention strategies. The burden of childhood obesity disproportionally affects marginalized populations [1–3]. Disparities in obesity prevalence are already present by preschool age [4], suggesting that they may originate in the earliest stages of life. Numerous studies [5–8] suggest that the period from conception to age 2 years, also known as the first 1000 days, could be a critical period for the development of childhood obesity.

One pervasive environmental exposure of concern is particulate air pollution, which has been associated with adverse fetal outcomes [9–13] and lower birth weight [14, 15]. Prenatal air pollution has also been associated with higher risk for childhood obesity [16–18]. One explanation for this apparent paradox is the fetal programming hypothesis [19], which holds that a maladaptive intrauterine environment leads to an adaptive response that alters the fetal metabolic and hormonal milieu designed for intrauterine survival. Thus, growth restriction in utero is associated with greater catch-up growth in infancy [20] and risk of obesity later in life [21–24].

However, epidemiological findings between prenatal air pollutant exposures and fetal and infant growth have been mixed [25–33]. For example, prenatal exposure to black carbon has been linked to reduced fetal growth in the U.S. birth cohort, Project Viva (*n* = 1597) [27]. Similar inverse associations were observed for PM_2.5_ and its constituents in a Chinese cohort (*n* = 4319) [28] and for PM_10_, PM_2.5_, and NO_2_ in a Scotland cohort (*n* = 13,775) [29]. Higher levels of air pollution exposure during pregnancy were associated with slower infant growth in a rural Ghanaian pregnancy cohort (*n* = 1414) [30] and lower weight in males after age 2 years from a U.S. cohort (*n* = 4797) [31]. Conversely, higher prenatal NO_2_ exposure was linked to a faster growth rate from birth to early childhood in a Chinese birth cohort (*n* = 5752) [32]. In another Chinese cohort (*n* = 62,540) [33], Tan et al. found that increased exposure to air pollutants during pregnancy was associated with a higher risk of being in either a slow or rapid BMI trajectory. Often, these studies assessed growth measurements within 12 months after birth, which may not capture the entire critical period for the development of childhood obesity [5]. Additionally, most of these studies only assess growth at the beginning of the study and at the end of follow-up, which may not account for the rapid change in growth rate during the first few months of life. No studies have evaluated in utero growth measures combined with postnatal weight trajectories along a continuum among marginalized populations, who have disproportionately high levels of obesity risk [34].

Longitudinal studies with greater density of growth data are needed, especially among marginalized populations, to fully assess the effect of exposure to air pollutants during pregnancy and fetal and infant growth. This study evaluated associations between prenatal exposure to ambient air pollution and individual weight trajectories from the 3rd trimester to 2 years of life among subjects from the Maternal and Development Risks from Environmental and Social Stressors (MADRES) study, a prospective pregnancy cohort that comprises a predominantly lower-income, Hispanic population in Los Angeles, California.

## Methods

### Study design and study population

This study was conducted as part of the MADRES study [35]. MADRES is a population-based prospective pregnancy cohort study that enrolled pregnant women in who met the following inclusion criteria: (1) less than 30 weeks gestation at the time of recruitment, (2) over 18 years of age, and (3) fluent English or Spanish speaker. Exclusion criteria were (1) HIV-positive status, (2) physical, mental, or cognitive disability that prevents participation or providing informed consent, (3) current incarceration, and (4) multiple gestations [35].

Beginning in November 2015, participants were recruited and then followed through pregnancy, at birth, and after birth through a series of in-person visits with interviewer-administered questionnaires, anthropometric measurements, and biospecimen collection. The MADRES study recruited participants primarily from prenatal healthcare clinics who provide healthcare services to medically disadvantaged populations [35]. Sources of recruitment include two non-profit community health clinics, one county hospital prenatal clinic, one private obstetrics and gynecology practice, and limited self-referrals [35]. As of September 2021, 6847 fetal and infant weight measurements were available for 710 children. For this analysis, children were excluded if they did not have (1) date-of-birth information, (2) a signed HIPAA authorization access to child medical record data, and (3) at least two weight measurements during the first 2 years of life. This process yielded a final sample size of 490 children with 6155 weight measurements (see Additional file 1, Figure S1-S2). An analysis was performed to compare the demographic characteristics of excluded children with only one weight measure and included children with at least two measures.

The study protocol was approved by the Institutional Review Board of the University of Southern California. Informed consent and HIPAA authorization to access medical records were obtained at study entry for each participant and her child.

### Air pollution assessment

Detailed residential histories were collected for each participant, beginning 2 years before pregnancy and throughout the follow-up period. Daily estimates of ambient air pollutants were assigned to individual residential locations using inverse distance squared weighted spatial interpolation based on data from the regulatory air monitoring stations (the United States Environmental Protection Agency Air Quality System). A time-weighting approach was applied to assess daily air pollution levels for subjects with more than one residence during pregnancy [35]. These pollutants include nitrogen dioxides (NO_2_), particulate matter < 10 µm (PM_10_), particulate matter < 2.5 µm (PM_2.5_), and ozone (O_3_). Daily ambient temperature and relative humidity estimates were obtained based on the gridmet 4 km × 4 km Abatzoglou model assigned based on residential location [36]. Pregnancy-averaged concentrations of air pollution and meteorological variables were calculated for each study subject based on the conception date which was estimated from gestational age at birth. Gestational age (GA) at birth was obtained from medical records or self-reported data using a hierarchy of methods [37–39]. GA was first assessed using a first trimester (< 14 weeks of gestation) ultrasound measurement of crown-rump length. If these data were unavailable, GA was assessed using a second trimester (< 28 weeks of gestation) ultrasound measurement of fetal biparietal diameter. If there were no ultrasound measurements in the 1st or 2nd trimester, GA was assessed based on a physician’s best clinical estimate from medical records. If none of the above data were available, GA was assessed based on participant’s self-reported last menstrual period. Postnatal air pollution concentrations were calculated as the mean concentrations from birth to the date when each weight measurement was collected.

### Weight assessment

Weight measurements were collected from both interviewer-administered questionnaires and each in-person visit. In utero estimates of fetal weight during the 3rd trimester were evaluated using head circumference (HC), abdominal circumference (AC), and femur length (FL) parameters from maternal ultrasound records using the Hadlock A formula [40]. Weight measures after birth were obtained using a combination of medical record abstractions from delivery and pediatric healthcare visits and study-staff measurements during in person follow-up visits at target ages (birth, 7–14 days, 1 months, 3 months. 6 months, 12 months, 18 months, and 24 months).

### Covariates

Several variables that are potentially relevant to infant growth were selected as covariates from the literature. Dagitty [41] was used to generate a directed acyclic graph (DAG) to assess the relationships between these variables (Figure S3). Covariates that were used to assess the effect of prenatal air pollution exposure on growth include maternal education level, maternal race, pre-pregnancy body mass index (BMI), hypertensive and diabetic disorders in pregnancy, recruitment site, maternal age at the time of study enrollment, ambient temperature during pregnancy, parity, postnatal air pollution concentration, and breastfeeding duration. Covariates were selected based on the minimal sufficient adjustment set suggested by Dagitty (maternal age, ambient temperature during pregnancy, pre-pregnancy BMI, recruitment site, education level, race/ethnicity) and prior literature (hypertensive disorder [42], breastfeeding duration [43, 44], gestational diabetes [45], and postnatal air pollution [30]). Information about maternal demographic variables was self-reported from interviewer-administered questionnaires. Maternal pre-pregnancy BMI was calculated as self-reported pre-pregnancy weight in kilograms divided by standing height in meters squared at the first study visit (< 30 weeks of gestation) by a study staff member. Information about maternal hypertensive disorders (HTN) and diabetic disorders (DD) was collected from physician diagnoses or lab results from maternal medical records (HTN: 97.7%, DD: 95%) or self-report (HTN: 2.3%, DD: 5%). Gestational hypertension was defined as an elevation of diastolic blood pressure of 90 mm Hg or more or systolic blood pressure of 140 mmHg or more at least on two consecutive prenatal visits abstracted from records. Physician-diagnosed gestational hypertension data were used if available. If unavailable, blood pressure measurements during pregnancy were used. If data from both sources are unavailable, self-reported hypertension diagnosis data was used. Physician-diagnosed gestational diabetes data were used if available. If unavailable, glucose challenge test (GCT) results and oral glucose tolerance test (OGTT) results from electronic medical records were used to define gestational diabetes. Self-reported gestational diabetes diagnosis data were used if data from both GCT and OGTT were missing. Information about current breastfeeding status was collected during the follow-up visits at 3, 6, and 12 months. Breastfeeding duration was categorized as less than 6 months, 6 to 12 months, and more than 12 months.

### Statistical analyses

Univariate analyses and visualizations were conducted to check for missing or implausible values of weight between the 3rd trimester to 2 years of age. The non-linear relationship between log-transformed weight and age was assessed using linear spline modeling with several knot points [46]. Several models were fitted with knot positions at several prespecified time points (at birth, 10 days, 3 months, 6 months, 9 months, and 12 months), which are consistent with the knot points identified from a previous study [46]. Model fit (R2) was assessed using different numbers of knots and degrees and summarized in Table S1. The increase in R2 was trivial when the number of knots and degrees was increased (0.893 [knot = 1; degree = 1]—0.906 [knot = 5; degree = 3]). Therefore, we selected one knot point representing early infancy (3 months) and another knot point representing late infancy (6 months). The linear model was chosen over the polynomial model to ensure a more straightforward interpretation of the results, while maintaining a comparable level of model fit. Our linear mixed effect models assumed random intercept and random slope for each subject. Statistical interactions between each air pollutant and each spline term were tested to assess the different effects of exposure to air pollution on weight trajectories across all spline periods.

We used two piecewise spline models to assess the effect of air pollutants on weight, overall growth, and growth during each time period [47]. To assess the effect of air pollutants on overall growth, we used the model (1), where *B* represents the overall slope, *t* represents to time from the 3rd trimester to age 2 years, and the two *D*’s parameterize the difference between the overall slope *B* and the spline. The two τ terms were constructed based on the 3 linear pieces (t1–t3) [47].

1 $$\mathrm{Y}=\mathrm{a}+{\mathrm{B}}^{*}\mathrm{t}+\mathrm{d}{1}^{*}\uptau 1+\mathrm{d}{2}^{*}\uptau 2$$

To test the effect of air pollutants on growth during each time period, we reparametrized model (1) to model (2) listed below, where B1, B2, and B3 represent the slopes of the 3 linear pieces (t1: 3rd trimester to 3 months of age; t2: 3 months of age to 6 months of age; t3: 6 months of age to 24 months of age).

2 $$\mathrm{Y }=\mathrm{ a }+\mathrm{ B}{1}^{*}\mathrm{t}1 +\mathrm{ B}{2}^{*}\mathrm{t}2 +\mathrm{ B}{3}^{*}\mathrm{t}3$$

Two main mixed effect models were used in the analysis. Model 1 included age, two age-related spline terms, each air pollutant separately, and interactions between air pollutant and all three age terms. Model 2 included all terms in Model 1 but further adjusted for covariates listed above. Multipollutant models were also performed that adjusted for O_3_ or NO_2_ in model 1 and model 2. All air pollutant levels were treated as continuous variables and were normalized using z-score standardization [z-score = (pollutant concentration-mean concentration) /standard deviation] [48]. All results were scaled to compare a change in pollutant from the 10th percentile to the 90th percentile of exposure. For instance, for PM_2.5_, we evaluated the effect on growth of a change in concentration from 10.5 to 13.4 μg/m^3^. Variance inflation factor (VIF) was obtained from single pollutant models to test multicollinearity and there were no VIF greater than 10 for any covariates except for the air pollutant and the interaction between pollutant and the splines, indicating the multicollinearity of covariates in our models are moderate and acceptable. We performed two sensitivity analyses to further investigate the impact of prenatal air pollution on offspring growth trajectory. The first analysis assessed the effect stratified by the infant sex. The second analysis excluded subjects with preterm birth to examine the robustness of our findings. The significance threshold was 0.05 and all tests were two-sided. Analyses were performed using SAS version 9.4 (SAS Institute).

## Results

### Characteristics of the mother-infant pairs

We compared characteristics of our infants with at least two weight data points (*n* = 490) to infants having only one weight datapoint (*n* = 220) (Table 1) and did not find significant differences except for maternal age and distribution of race/ethnicity. Maternal participants self-identified predominantly as Hispanic (79.8%), were predominantly low-income (defined as annual household income < $50,000) with an average age of 29 years at consent, and an average pre-pregnancy BMI of 28.95 kg/m^2^. The majority of the population was overweight (25 < BMI < 30) (31.5%) or obese (BMI ≥ 30) (38.3%) prior to pregnancy. The mean gestational age at birth was 39 weeks and the mean (standard deviation, SD) birth weight was 3290 (500) grams. On average, there were 12 (range: 2–42) weight measures for each child. The mean (SD) follow-up time was 146 (174) days. The weight measurements were obtained from 27 weeks gestation until 24 months after birth.

**Table 1:** Characteristics of eligible mothers and infants with only one weight measure and included in the study

Maternal and infant characteristics	At least one weight (*n* = 710)	At least two weight (*n* = 490)	Only one weight (*n* = 220)	*P* value
	**No. (%), mean (SD)**	**No. (%), mean (SD)**	**No. (%), mean (SD)**	
Maternal age (at consent)	28 (6.0)	29 (6.1)	27 (5.4)	< 0.01
Recruitment site				
LAC + USC	132 (18.6)	83 (16.9)	49 (22.2)	0.09
Eisner	492 (69.3)	351 (71.6)	141 (64.1)	
USC Obstetrics and Gynecology	57 (8.0)	41 (8.4)	16 (7.3)	
Community Recruit	5 (0.7)	3 (0.6)	2 (0.9)	
South Central Clinic	24 (3.4)	12 (2.5)	12 (5.5)	
Race/ethnicity				
White (non-Hispanic)	37 (5.5)	30 (6.2)	7 (3.8)	0.02
Black (non-Hispanic)	85 (12.7)	56 (11.5)	29 (15.8)	
Hispanic	523 (78.1)	388 (79.8)	135 (73.4)	
Multiracial, non-Hispanic	9 (1.3)	4 (0.8)	5 (2.7)	
Other, non-Hispanic	16 (2.4)	8 (1.7)	8 (4.3)	
Maternal education				
< 12th Grade	172 (25.7)	120 (24.6)	52 (28.4)	0.71
High school	209 (31.2)	150 (30.8)	59 (32.2)	
Some college	179 (26.7)	132 (27.1)	47 (25.7)	
Complete college	73 (10.9)	56 (11.5)	17 (9.3)	
Graduate training	37 (5.5)	29 (6)	8 (4.4)	
Household Income				
Less than $15,000	138 (20.6)	91 (18.7)	47 (25.5)	0.17
$15,000 to $29,999	163 (24.3)	118 (24.2)	45 (24.5)	
$30,000 to $49,999	76 (11.3)	57 (11.7)	19 (10.3)	
$50,000 to $99,999	38 (5.7)	25 (5.1)	13 (7.1)	
$100,000 or more	34 (5.1)	29 (6.0)	5 (2.7)	
Do not know	222 (33.1)	167 (34.3)	55 (29.9)	
Diabetic disorders				
Normal	460 (66.5)	323 (66.6)	137 (66.2)	0.33
Glucose intolerant	136 (19.7)	94 (19.4)	42 (20.3)	
Gestational diabetes mellitus	62 (9.0)	40 (8.3)	22 (10.6)	
Chronic diabetes	34 (4.9)	28 (5.8)	6 (2.9)	
Hypertensive disorders				
Normal	551 (78.5)	388 (79.4)	163 (76.5)	0.74
Preeclampsia-eclampsia	65 (9.2)	42 (8.6)	23 (10.8)	
Chronic hypertension	26 (3.7)	16 (3.3)	10 (4.7)	
Chronic hypertension with preeclampsia	16 (2.3)	12 (2.5)	4 (1.9)	
Gestational hypertension	44 (6.3)	31 (6.3)	13 (6.1)	
Pre-pregnancy BMI				
Underweight (BMI < 18.5)	18 (2.6)	11 (2.2)	7 (3.2)	0.38
Normal (BMI of 18.5 to < 25)	212 (30)	137 (28.0)	75 (34.5)	
Overweight (BMI of 25 to < 30)	218 (30.8)	154 (31.5)	64 (29.5)	
Class 1 Obese (BMI of 30 to < 35)	151 (21.4)	107 (21.8)	44 (20.3)	
Class 2 Obese (BMI of 35 to < 40)	63 (8.9)	49 (10.0)	14 (6.5)	
Class 3 Obese (BMI of 40 or higher)	45 (6.4)	32 (6.5)	13 (6.0)	
Birth weight (g)	3260 (500)	3290 (500)	3210 (500)	0.08
Gestational age at birth (weeks)	39 (1.8)	39 (1.8)	39 (1.8)	0.70

*SD* Standard deviation

### Air pollution concentrations

Pregnancy-averaged concentrations of ambient air pollutants at the maternal residence are summarized in Table 2. The mean(SD) 24-h averages of PM_2.5_, PM_10_, NO_2_, and O_3_ were 12.0 (1.1) μg/m^3^, 28.5 (4.7) μg/m^3^, 16.4 (2.9) ppb, and 26.2 (2.9) ppb, respectively. Concentrations of the air pollutants from 490 mothers in our study were lower compared to the concentrations of the 220 mothers with only one weight (see Additional file 1, Table S2). Correlations of air pollutant levels and meteorological factors are summarized in Additional file 1: Figure S4 (see Additional file 1). PM_2.5_ was positively correlated with PM_10_ (*r* = 0.65) and NO_2_ (*r* = 0.49). NO_2_ was negatively correlated with O_3_ (*r* =  − 0.74).

**Table 2:** Distribution of mean air pollutants and meteorological factors during pregnancy (*n* = 490)

Air pollutant	*N*	Min	Median	IQR	Mean	SD	10th Percentile	90th Percentile	Max
PM_2.5_ (μg/m^3^)	489	7.6	11.9	1.7	12.0	1.1	10.5	13.4	17.6
PM_10_ (μg/m^3^)	489	18.1	28.2	7.5	28.5	4.7	22.4	34.7	45.2
NO_2_ (ppb)	489	8.2	16.5	3.7	16.4	2.9	12.9	20.4	24.5
O_3_ (ppb)	489	19.0	25.9	4.2	26.2	2.9	23.1	29.6	41.5
Temperature (°C)	489	10.1	19.3	1.8	19.2	1.4	17.4	20.7	22.5
Relative humidity (%)	489	42.8	62.1	5.4	61.6	4.1	56.3	66.4	70.9

*IQR* Interquartile range, *SD* Standard deviation

### Ambient air pollution and infant growth

The estimated growth in weight per year of age was significantly higher (1.08%, 95%CI [1.00%, 1.16%]) comparing a change in PM_2.5_ from the 10th percentile to the 90th percentile (Table 3). However, this trend did not remain significant in Model 2 (the adjusted model). Significant interactions between exposure and the spline term representing the period from the 3rd trimester to age 3 months were observed for PM_2.5_, NO_2_, and O_3_ in Model 1 (the unadjusted model) and Model 2. Comparing a change in pollutant from the 10th percentile to the 90th percentile, the estimated growth in weight was 1.55% (95%CI 1.20%, 1.99%) and 1.64% (95%CI 1.27%, 2.13%) higher for PM_2.5_ and NO_2_, while 0.68% (95%CI 0.54%, 0.86%) lower for O_3_ in Model 1. The estimated growth from age 6 months to age 2 years was 0.90% (95%CI 0.82%, 1.00%) lower comparing a change in NO_2_ from the 10th percentile to the 90th percentile. The differences in growth by air pollutant levels were similar in Model 2 and in multipollutant models (see Additional file 1, Table S3). No other statistically significant interactions were observed.

**Table 3:** Percent difference in growth comparing change in pregnancy-averaged levels of air pollutants from 10 to 90th percentile (single-pollutant models)

		**Model 1**				**Model 2**			
		**Percent difference in growth**	**95% CI**		***P* value**	**Percent difference in growth**	**95% CI**		***P* value**
PM_2.5_	3rd trimester to 2 years	*1.08*	*1.00*	*1.16*	*0.04*	1.06	0.99	1.14	0.09
	3rd trimester to 3 months	*1.55*	*1.20*	*1.99*	< *0.01*	*1.51*	*1.14*	*1.99*	< *0.01*
	3 months to 6 months	1.18	0.85	1.62	0.32	1.13	0.88	1.45	0.33
	6 months to 2 years	0.94	0.85	1.04	0.24	0.94	0.86	1.01	0.11
PM_10_	3rd trimester to 2 years	1.00	0.92	1.08	0.94	0.96	0.89	1.04	0.30
	3rd trimester to 3 months	1.16	0.89	1.51	0.28	1.02	0.76	1.38	0.87
	3 months to 6 months	1.19	0.84	1.69	0.32	1.13	0.86	1.49	0.37
	6 months to 2 years	0.92	0.81	1.04	0.19	0.91	0.83	1.00	0.06
NO_2_	3rd trimester to 2 years	1.04	0.97	1.12	0.25	1.04	0.96	1.11	0.34
	3rd trimester to 3 months	*1.64*	*1.27*	*2.13*	< *0.01*	*1.56*	*1.17*	*2.07*	< *0.01*
	3 months to 6 months	1.00	0.72	1.39	0.99	1.03	0.80	1.33	0.82
	6 months to 2 years	*0.90*	*0.82*	*1.00*	*0.04*	*0.90*	*0.84*	*0.98*	*0.01*
O_3_	3rd trimester to 2 years	0.98	0.92	1.05	0.64	0.95	0.89	1.02	0.15
	3rd trimester to 3 months	*0.68*	*0.54*	*0.86*	< *0.01*	*0.60*	*0.46*	*0.78*	< *0.01*
	3 months to 6 months	1.08	0.80	1.46	0.61	1.10	0.87	1.40	0.43
	6 months to 2 years	1.10	1.00	1.21	0.06	*1.09*	*1.01*	*1.17*	*0.03*

Model 1 includes infant age, two spline terms, and interactions between air pollutants and age and spline terms. Model 2 further adjusted for maternal education level, maternal race, pre-pregnancy BMI, hypertensive disorders, diabetic disorders, recruitment site, maternal age, parity, breastfeeding duration, postnatal air pollutant (same as the exposure), and ambient temperature

*CI* confidence interval

*P* values < 0.05 were italicized

### Ambient air pollution and infant attained weight

**Fig. 1:**
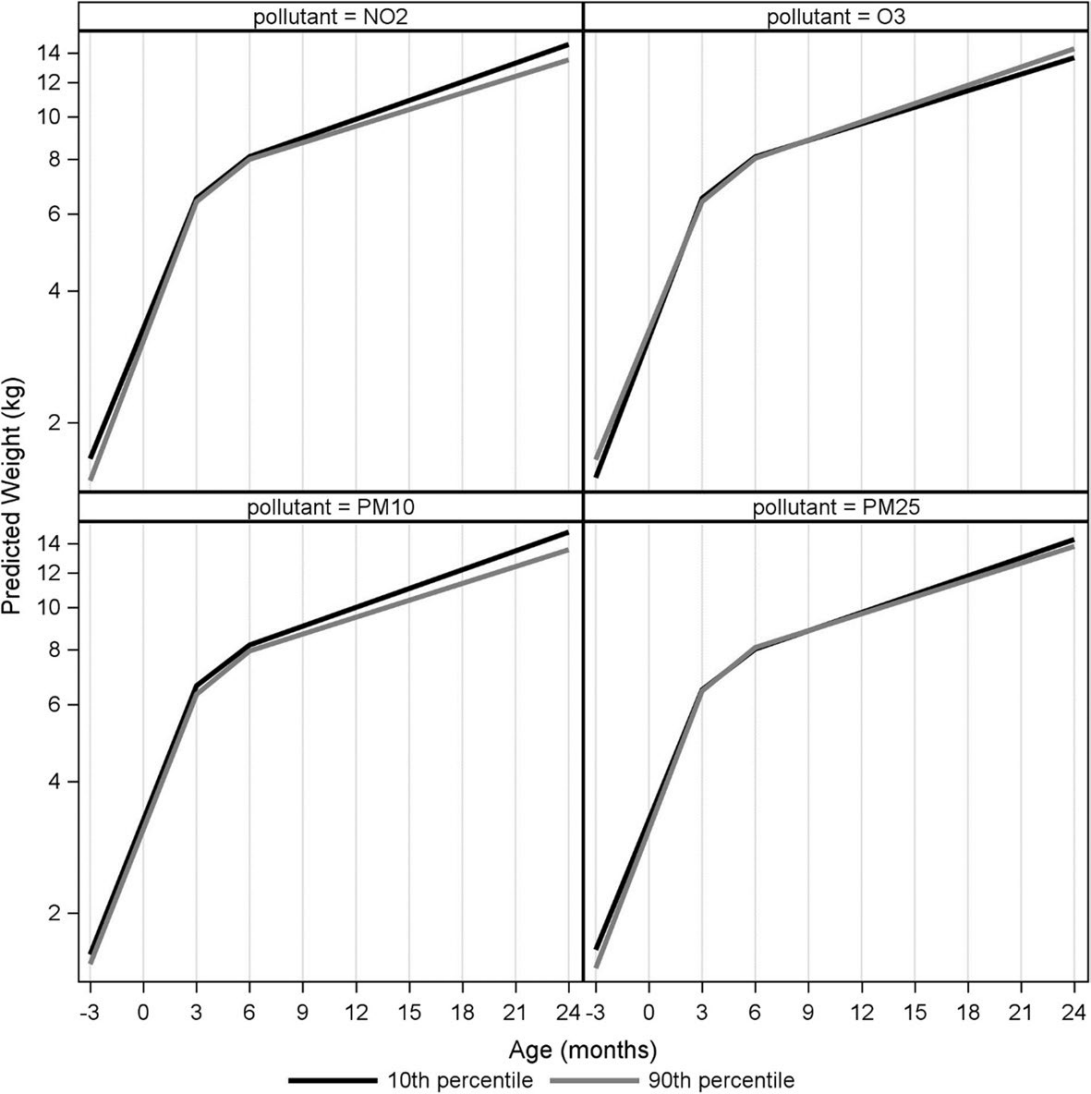
Infant averaged weight trajectory by ambient air pollutant levels. The infant weight trajectory was assessed using piecewise spline models with knots at 3 and 6 months of age. Log weight was analyzed, and results are presented with back-transformed values on a natural log-scaled axis. Weight trajectories are depicted for prenatal air pollution levels at the 10th and 90th percentiles, shown as black and grey lines, respectively. Higher prenatal exposure to PM_2.5_, PM_10_, and NO_2_ were associated with decreased weight at the 3rd trimester, at birth, and at age 2 years. The estimated growth from age 6 months to age 2 years was lower comparing a change in NO_2_ from the 10th percentile to the 90th percentile

Figure 1 shows the infant’s averaged weight trajectory by ambient air pollutant levels. On average, infants gained 11.9 kg from the 3rd trimester to 2 years of age. Comparing a change in pollutant from the 10th percentile to the 90th percentile, the percent difference in weight at 2 years was − 7.50% (95% CI − 13.57%, − 1.02%) for PM_10_ and − 7.00% (95% CI − 11.86%, − 1.88%) for NO_2_ in model 1 (Table 4). The significant inverse associations for NO_2_ and PM_10_ remained when we adjusted for covariates in Model 2 and when we further adjusted for O_3_ (see Additional file 1, Table S4). We also observed significant positive associations between O_3_ exposure and estimated weight at age 2 years; however, this association was attenuated to the null when we further adjusted for NO_2_ in a multipollutant model.

**Table 4:** Percent difference in mean weight comparing change in pregnancy-averaged levels of air pollutants from 10 to 90th percentile (single-pollutant models)

**Air pollutant**	**Age**	**Model 1**				**Model 2**			
		**Percent difference in weight**	**95% CI**		***P* value**	**Percent difference in weight**	**95% CI**		***P* value**
PM_2.5_	3rd trimester	*-10.24*	*-15.00*	*-5.21*	< *0.01*	*-9.25*	*-14.72*	*-3.43*	< *0.01*
	birth	*-5.77*	*-9.45*	*-1.93*	< *0.01*	*-5.01*	*-9.04*	*-0.81*	*0.02*
	2 years	-3.32	-8.52	2.18	0.23	-3.58	-8.09	1.16	0.14
PM_10_	3rd trimester	*-7.23*	*-12.29*	*-1.88*	*0.01*	-4.93	-11.08	1.64	0.14
	birth	*-5.72*	*-9.45*	*-1.83*	< *0.01*	*-4.68*	*-9.06*	*-0.08*	*0.05*
	2 years	*-7.50*	*-13.57*	*-1.02*	*0.02*	*-8.85*	*-14.13*	*-3.25*	< *0.01*
NO_2_	3rd trimester	*-10.85*	*-15.67*	*-5.74*	< *0.01*	*-10.94*	*-16.44*	*-5.07*	< *0.01*
	birth	*-5.78*	*-9.54*	*-1.88*	< *0.01*	*-6.45*	*-10.47*	*-2.24*	< *0.01*
	2 years	*-7.00*	*-11.86*	*-1.88*	*0.01*	*-7.79*	*-12.00*	*-3.38*	< *0.01*
O_3_	3rd trimester	*5.72*	*0.67*	*11.02*	*0.03*	*9.97*	*3.84*	*16.45*	< *0.01*
	birth	1.26	-2.27	4.92	0.49	3.91	-0.11	8.09	0.06
	2 years	4.07	-1.18	9.59	0.13	*4.82*	*0.19*	*9.65*	*0.04*

Model 1 includes infant age, two spline terms, and interactions between air pollutants and age and spline terms. Model 2 further adjusted for maternal education level, maternal race, pre-pregnancy BMI, hypertensive disorders, diabetic disorders, recruitment site, maternal age, parity, breastfeeding duration, postnatal air pollutant (same as the exposure), and ambient temperature

*CI* confidence interval

*P* values < 0.05 were italicized

We observed similar significant associations between prenatal ambient air pollution exposure and reduced weight at the 3rd trimester and at birth (Table 4). Comparing a change in pollutant from the 10th percentile to the 90th percentile, the percent difference in weight at the 3rd trimester was − 10.24% (95% CI − 15.00%, − 5.21%) for PM_2.5_, − 7.23% (95% CI − 12.29%, − 1.88%) for PM_10_, − 10.85% (95% CI − 15.67%, − 5.74%) for NO_2_ and 5.72% (95% CI 0.67%, 11.02%) for O_3_ in Model 1. Comparing a change in pollutant from the 10th percentile to the 90th percentile, the percent difference in weight at birth was − 5.77% (95% CI − 9.45%, − 1.93%) for PM_2.5_, − 5.72% (95% CI − 9.45%, − 1.83%) for PM_10_ and − 5.78% (95% CI − 9.54%, − 1.88%) for NO_2_. The percent differences in weight at the 3rd trimester and at birth by air pollutant levels were similar in Model 2 and in multipollutant models (see Additional file 1, Table S4).

We conducted a sensitivity analysis to test the effect of air pollution on growth trajectory stratified by baby’s sex (Table S5.1-S5.4). Similar to the results from the main analysis, PM_2.5_ and NO_2_ were associated with decreased weight at the 3rd trimester for both male and female fetuses while both pollutants were associated with decreased weight at age 2 years for female children only. Consistent with the main analysis, we observed significant positive effects of prenatal NO_2_ exposure and growth from the 3rd trimester to age 3 months among male children. There were nonsignificant positive associations between PM_2.5_ and growth from 3rd trimester to age 3 months among male and female children.

Our study included 49 (10%) pre-term births. We conducted a sensitivity analysis to exclude subjects with preterm birth (Table S6-S7). Consistent with the findings from the main analysis, we found positive associations between PM2.5, NO2, and growth from the 3rd trimester to age 3 months and negative associations between NO2 and growth from 6 months to age 2 years. Prenatal exposure to NO2 and PM10 were inversely associated with weight at age 2 years, which is similar to the results in the main analysis.

## Discussion

In this pregnancy cohort study that comprises a low-income, primarily Hispanic population, prenatal exposures to ambient NO_2_, PM_2.5_, and PM_10_ were associated with lower attained weight at the 3rd trimester, at birth, and persisted at age 2 years in some cases. However, higher levels of prenatal exposure to PM_2.5_ and NO_2_ were associated with increased growth from the 3rd trimester to age 3 months, suggesting some pollutants may contribute to “catch-up growth” in early infancy, whereas higher exposure to NO_2_ was also associated with decreased growth from age 6 months to age 2 years. Our results suggest a complicated and dynamic exposure-weight relationship in which higher prenatal exposures are associated with faster growth rates in early infancy but lower overall attained weight by age 2.

Our findings add to a growing literature supporting an association between prenatal air pollution exposure and altered fetal and infant growth [49–53]. Our findings are consistent with a recent meta-analysis [49] indicating that increased prenatal exposure to NO_2_ was associated with estimated fetal weight (EFW) restriction in five out of six studies. Consistent with our results, one cohort study in China [50] has suggested that prenatal exposure to PM_2.5_ and PM_10_ was associated with decreased EFW z-score. However, this contrasts with the findings in a Netherlands [51] cohort study where elevated exposure to PM_10_ was associated with increased EFW in mid-pregnancy. Our results are also consistent with results from Project Viva [52], in which Fleisch et al. reported a lower birth weight-for-gestational age z-score (an indicator of fetal growth) among infants exposed to the highest (vs. lowest) quartile of black carbon during the 3rd trimester.

We also found that the growth rate from the 3rd trimester to 3 months of age was steeper for babies with higher prenatal exposure to PM_2.5_ and NO_2_, which is consistent with a majority of other studies. For instance, the Southern California Mother’s Milk Study [54] found that the growth rate from 1 to 6 months of age was steeper for babies with higher prenatal exposure to NO_2_. In Project Viva [52], Fleisch et al. found that infants exposed to the highest (vs. lowest) quartile of neighborhood traffic density during pregnancy had more rapid increase in weight-for-length z-score from birth to 6 months of age. A longitudinal study in Spain [55] observed stronger positive associations between early life exposure to PM_10_ and NO_2_ and BMI growth from 0–2 months relative to other time periods from 2 months to 5 years of age.

The existing studies on prenatal air pollutant exposures and infant growth measures are inconsistent although the generally accepted hypothesis is that air pollutant exposures increase risk of childhood obesity [56]. In contrast to this hypothesis, we found that prenatal exposure to ambient NO_2_ and PM_10_ during pregnancy was associated with lower attained weight for babies at 2 years of age. Similar to our results, a Spanish birth cohort study [57] observed an inverse association between prenatal NO_2_ exposure and infant weight at 1 year. In a Chinese cohort [33], Tan et al. found that higher prenatal exposure to PM_10_ was associated with a higher risk of slow BMI trajectory relative to normal BMI trajectory from birth to age of 6. A Korean birth cohort study [58] revealed that persistent low weight-for-height percentile trajectory was associated with the highest quartile of PM_2.5_ exposure during mid-pregnancy.

However, many other studies have shown an increased risk for childhood obesity or more rapid growth from prenatal pollutant exposures, though the exposures evaluated have varied. In the Project Viva cohort [52], Fleisch et al. showed that infants exposed to the highest quartile of neighborhood traffic density had higher odds of weight-for-length ≥ 95th percentile at 6 months. The same group [59] also demonstrated a positive association between proximity to major roadways at birth (< 50 m) and fat mass at mid-childhood (median age 7.7 years). One Chinese cohort [60] reported an increase in weight-for-length and BMI z-score for 1-year-old children exposed to higher levels of PM_2.5_ during pregnancy.

Several possible explanations may account for discrepancies in results across the literature. One reason could be that air pollution-related obesity risk develops over a longer period than 2 years of life and we did not follow our children’s growth long enough in our cohort. Other reasons include differences in geographic location of study participants, the chemical composition of air pollutants [60, 61], differences in modeling approaches, selected exposure time windows [61], and the demographic characteristics of the study population.

During pregnancy, women experience increased alveolar ventilation rate, leading to higher uptake of inhaled air pollutants [62]. Small-sized particles can penetrate the alveolar–capillary barrier and enter the maternal bloodstream, reach and across the placenta, and reach the fetus [63–65]. The hypothesized biological mechanisms involved in air pollution-related offspring growth include induction of maternal, placental, or fetal oxidative stress, DNA damage, inflammation, vascular dysfunction, and altered mitochondrial function [13, 33, 66, 67]. These mechanisms can impede nutrient transfer from mother to fetus and disrupt the fetal metabolic and endocrine systems [68–71]. For instance, prenatal exposure to traffic-related air pollution has been associated with higher cord blood leptin and high molecular weight adiponectin levels, which affect glucocorticoid levels and childhood weight gain [68]. Prenatal air pollutant exposures have been associated with alterations in maternal and neonate thyroid hormone levels as well, suggesting a potential for endocrine-disrupting effects [69–71].

There are several strengths in this study. First, this large, prospective cohort study is comprised primarily of a lower-income, Hispanic population, which has a disproportionally larger burden of childhood obesity [72] and higher levels of exposure to air pollutants [73] relative to the non-Hispanic white population. Therefore, our findings help to better understand the role of exposure to environmental factors in the context of disparities in growth patterns and obesity risk in marginalized populations which have historically been left out of research studies. Second, numerous repeated measurements of weight were used to assess the individual growth trajectories for infants before 2 years of age. This improves upon existing studies that collected weight measurements only at limited time points. Third, our study evaluated in utero growth measures and postnatal weight trajectories along a continuum which provides a more complete picture of how prenatal exposure to air pollutants affects early life growth. Lastly, we adjusted for postnatal air pollutant exposures as well as evaluated multipollutant models to try to more fully understand the impacts of each pollutant.

Our study also has limitations. To evaluate growth on a continuum and bridge fetal and postnatal time periods, we had to use two different methods for assessing weight. We used the Hadlock A formula [40] to estimate fetal weight, whereas in the postnatal period, we used measured weight of the infant directly. A recent systematic review [74] suggested that the Hadlock A formula produced the most accurate results, with the lowest levels of random error, relative to other commonly used methods for the estimation of fetal weight. That said, using ultrasound records to estimate fetal measurements likely has a greater level of measurement error than measuring birthweight in infants directly. While we did conduct multi-pollutant modeling, we did not examine the cumulative effects of the four ambient air pollutants together using a mixtures analysis in part due to statistical challenges in incorporating formal mixtures modeling with growth trajectory modeling. Less than 2% of mothers reported smoking or consuming alcohol during pregnancy, thus preventing us from adjusting for these variables in our analyses. Because the children in MADRES are still young, we did not have the ability to extend the growth trajectories to older ages, which could give a more complete picture of the lasting effects of air pollutant exposures on weight gain. In the future, as the cohort ages, we will be able to extend the follow-up period to later childhood and adolescence. Lastly, one of the next steps will be to use more sophisticated modeling approaches in our trajectory modeling to identify more refined sensitive windows of exposure to air pollutants during pregnancy.

## Conclusions

Our study provides evidence that higher prenatal exposure to PM_2.5_ and NO_2_ were associated with an increased growth rate from the 3rd trimester to 3 months after birth and a reduced attained weight at late pregnancy and age 2 years in infants living in the urban Los Angeles area. Our findings have important public health implications. Our results suggest pregnancy as a potential critical period for implementing interventions aimed at reducing personal exposure to ambient air pollution. These interventions are essential for mitigating the impact of air pollution on offspring growth and promoting healthy development. Exposure to air pollution during pregnancy is a modifiable risk factor and efforts to reduce ambient exposures will continue to be important particularly in vulnerable populations.

### Supplementary Information

**Additional file 1: Table S1.** Summary of Model Fit with Different Numbers of Knots and Degrees.** Table S2.** Comparison of Pregnancy-averaged Air Pollution Concentration and Meteorological Factors between Subjects with Only One Weight Measurement (*n*=220), and with At least Two Measurements (*n*=490). **Table S3.** Percent Difference in growth Comparing Change in Pollutant from 10th to 90th Percentile (Multipollutant Models). **Table S4.** Percent Difference in Mean Weight Comparing Change in Pollutant from 10^th^ to 90^th^ Percentile (Multipollutant Models). **Table S5.1.** Percent Difference in growth Comparing Change in Pregnancy-averaged Levels of Air Pollutants from 10th to 90th Percentile (Single-Pollutant Models) for Males. **Table S5.2.** Percent Difference in growth Comparing Change in Pregnancy-averaged Levels of Air Pollutants from 10^th^ to 90^th^ Percentile (Single-Pollutant Models) for Females. **Table S5.3.** Percent Difference in Mean Weight Comparing Change in Pregnancy-averaged Levels of Air Pollutants from 10^th^ to 90^th^ Percentile (Single-Pollutant Models) for Males. **Table S5.4.** Percent Difference in Mean Weight Comparing Change in Pregnancy-averaged Levels of Air Pollutants from 10^th^ to 90^th^ Percentile (Single-Pollutant Models) for Females. **Table S6.** Percent Difference in Growth Comparing Change in Pregnancy-averaged Levels of Air Pollutants from 10^th^ to 90^th^ Percentile (exclude subjects with pre-term birth). **Table S7.** Percent Difference in Mean Weight Comparing Change in Pregnancy-averaged Levels of Air Pollutants from 10^th^ to 90^th^ Percentile (exclude subjects with pre-term birth). **Figure S1.** Conceptual Flow Chart. **Figure S2.** Spaghetti Plot of Weight. **Figure S3**. Directed acyclic graph. **Figure S4.** Correlation Matrix for Air Pollutants and Meteorological factors.

## Data Availability

The datasets used and/or analyzed during the current study are available from the corresponding author on reasonable request.

## Abbreviations

AC: Abdominal circumference
BMI: Body mass index
CI: Confidence interval
DD: Diabetic disorders
EFW: Estimated fetal weight
FL: Femur length
GA: Gestational age
GCT: Glucose challenge test
HC: Head circumference
HTN: Hypertensive disorders
MADRES: Maternal and Developmental Risks from Environmental and Social Stressors
NO_2_: Nitrogen dioxide
O_3_: Ozone
OGTT: Oral glucose tolerance test
PM_10_: Particulate matter < 10 µm
PM_2.5_: Particulate matter < 2.5 µm
