# Supplementary material for: Prenatal ambient air pollution exposure and child weight trajectories from the 3rd trimester of pregnancy to 2 years of age: a cohort study
Source: BMC Med. 2023 Sep 7;21:341. doi: 10.1186/s12916-023-03050-y (PMC10483706; doi:10.1186/s12916-023-03050-y)
Supplement: Supplementary file 1 — Additional file 1: Table S1. Summary of Model Fit with Different Numbers of Knots and Degrees. Table S2. Comparison of Pregnancy-averaged Air Pollution Concentration and Meteorological Factors between Subjects with Only One Weight Measurement (n=220), and with At least Two Measurements (n=490). Table S3. Percent Difference in growth Comparing Change in Pollutant from 10th to 90th Percentile (Multipollutant Models). Table S4. Percent Difference in Mean Weight Comparing Change in Pollutant from 10th to 90th Percentile (Multipollutant Models). Table S5.1. Percent Difference in growth Comparing Change in Pregnancy-averaged Levels of Air Pollutants from 10th to 90th Percentile (Single-Pollutant Models) for Males. Table S5.2. Percent Difference in growth Comparing Change in Pregnancy-averaged Levels of Air Pollutants from 10th to 90th Percentile (Single-Pollutant Models) for Females. Table S5.3. Percent Difference in Mean Weight Comparing Change in Pregnancy-averaged Levels of Air Pollutants from 10th to 90th Percentile (Single-Pollutant Models) for Males. Table S5.4. Percent Difference in Mean Weight Comparing Change in Pregnancy-averaged Levels of Air Pollutants from 10th to 90th Percentile (Single-Pollutant Models) for Females. Table S6. Percent Difference in Growth Comparing Change in Pregnancy-averaged Levels of Air Pollutants from 10th to 90th Percentile (exclude subjects with pre-term birth). Table S7. Percent Difference in Mean Weight Comparing Change in Pregnancy-averaged Levels of Air Pollutants from 10th to 90th Percentile (exclude subjects with pre-term birth). Figure S1. Conceptual Flow Chart. Figure S2. Spaghetti Plot of Weight. Figure S3. Directed acyclic graph. Figure S4. Correlation Matrix for Air Pollutants and Meteorological factors. [file 12916_2023_3050_MOESM1_ESM.docx]

**Table S1.** Summary of Model Fit with Different Numbers of Knots and Degrees

| **knots** | **degree** | **R2** |
| --- | --- | --- |
| 1 | 1 | 0.893 |
| 1 | 2 | 0.896 |
| 1 | 3 | 0.905 |
| 2 | 1 | 0.904 |
| 2 | 2 | 0.906 |
| 2 | 3 | 0.905 |
| 3 | 1 | 0.905 |
| 3 | 2 | 0.906 |
| 3 | 3 | 0.906 |
| 4 | 1 | 0.906 |
| 4 | 2 | 0.907 |
| 4 | 3 | 0.907 |
| 5 | 1 | 0.907 |
| 5 | 2 | 0.907 |
| 5 | 3 | 0.906 |

| **Table S2.** Comparison of Pregnancy-averaged Air Pollution Concentration and Meteorological Factors between Subjects with Only One Weight Measurement (n=220), and with At least Two Measurements (n=490) | | | | | |
| --- | --- | --- | --- | --- | --- |
|  |  |  |  |  |  |
| **Air Pollutant** | **Only one weight (n=220)** | | **At least two weights (n=490)** | |  |
|  | **Mean** | **SD** | **Mean** | **SD** | **P value** |
| **PM_2.5_ (μg/m^3^)** | 12.3 | 1.2 | 12.0 | 1.1 | <0.01 |
| **PM_10_ (μg/m^3^)** | 30.7 | 4.7 | 28.5 | 4.7 | <0.01 |
| **NO_2_ (ppb)** | 17.6 | 3.0 | 16.4 | 2.9 | <0.01 |
| **O_3_ (ppb)** | 26.4 | 3.1 | 26.2 | 2.9 | 0.36 |
| **Temperature (°C)** | 19.4 | 1.3 | 19.2 | 1.4 | 0.06 |
| **Relative Humidity (%)** | 60.1 | 4.3 | 61.6 | 4.1 | <0.01 |

| **Table S3**. Percent Difference in growth Comparing Change in Pollutant from 10^th^ to 90^th^ Percentile (Multipollutant Models) | | | | | | | |
| --- | --- | --- | --- | --- | --- | --- | --- |
|  |  |  |  |  |  |  |  |
|  |  | **Model 1** | | | **Model 2** | | |
|  |  | **Percent Difference in Growth** | **95% CI** | | **Percent Difference in Growth** | **95% CI** | |
| **PM_2.5_*^a^*** | **3rd trimester to 2 years** | **1.08** | **1.00** | **1.16** | 1.06 | 0.99 | 1.14 |
|  | **3rd trimester to 3 months** | **1.55** | **1.20** | **1.99** | **1.51** | **1.14** | **1.99** |
|  | **3 months to 6 months** | 1.18 | 0.85 | 1.62 | 1.13 | 0.88 | 1.46 |
|  | **6 months to 2 years** | 0.94 | 0.85 | 1.04 | 0.93 | 0.86 | 1.01 |
| **PM_10_*^a^*** | **3rd trimester to 2 years** | 1.00 | 0.92 | 1.08 | 0.96 | 0.89 | 1.04 |
|  | **3rd trimester to 3 months** | 1.16 | 0.89 | 1.51 | 1.02 | 0.76 | 1.38 |
|  | **3 months to 6 months** | 1.19 | 0.84 | 1.69 | 1.13 | 0.86 | 1.49 |
|  | **6 months to 2 years** | 0.92 | 0.81 | 1.04 | 0.91 | 0.83 | 1.01 |
| **NO_2_*^a^*** | **3rd trimester to 2 years** | 1.04 | 0.97 | 1.12 | 1.04 | 0.97 | 1.11 |
|  | **3rd trimester to 3 months** | **1.65** | **1.27** | **2.13** | **1.56** | **1.17** | **2.08** |
|  | **3 months to 6 months** | 1.00 | 0.72 | 1.39 | 1.03 | 0.80 | 1.33 |
|  | **6 months to 2 years** | **0.90** | **0.82** | **1.00** | **0.91** | **0.84** | **0.98** |
| **O_3_*^b^*** | **3rd trimester to 2 years** | 0.98 | 0.92 | 1.05 | 0.95 | 0.89 | 1.01 |
|  | **3rd trimester to 3 months** | **0.68** | **0.54** | **0.85** | **0.60** | **0.46** | **0.78** |
|  | **3 months to 6 months** | 1.08 | 0.80 | 1.46 | 1.10 | 0.87 | 1.40 |
|  | **6 months to 2 years** | 1.09 | 0.99 | 1.20 | **1.08** | **1.00** | **1.16** |
| Model 1 includes infant age, two spline terms, interactions between air pollutants and age and spline terms, and O_3_*^a^* or NO_2_*^b^* | | | | | | | |
| Model 2 further adjusted for maternal education level, maternal race, pre-pregnancy BMI, hypertensive disorders, diabetic disorders, recruitment site, maternal age, parity, breastfeeding duration, postnatal air pollution, and ambient temperature | | | | | | | |

| **Table S4.** Percent Difference in Mean Weight Comparing Change in Pollutant from 10^th^ to 90^th^ Percentile (Multipollutant Models) | | | | | | | |
| --- | --- | --- | --- | --- | --- | --- | --- |
|  |  |  |  |  |  |  |  |
| **Air Pollutant** | **Age** | **Model 1** | | | **Model 2** | | |
|  |  | **Percent Difference in Weight (%)** | **95% CI** | | **Percent Difference in Weight (%)** | **95% CI** | |
| **PM_2.5_*^a^*** | **3rd trimester** | **-10.44** | **-15.28** | **-5.33** | **-8.87** | **-14.56** | **-2.80** |
|  | **birth** | **-5.98** | **-9.77** | **-2.02** | **-4.62** | **-8.96** | **-0.07** |
|  | **2 years** | -3.54 | -8.82 | 2.05 | -3.21 | -7.97 | 1.80 |
| **PM_10_*^a^*** | **3rd trimester** | **-7.22** | **-12.28** | **-1.86** | -4.90 | -11.05 | 1.69 |
|  | **birth** | **-5.70** | **-9.44** | **-1.81** | **-4.64** | **-9.03** | **-0.03** |
|  | **2 years** | **-7.52** | **-13.58** | **-1.03** | **-8.79** | **-14.10** | **-3.16** |
| **NO_2_*^a^*** | **3rd trimester** | **-14.36** | **-19.80** | **-8.55** | **-14.27** | **-20.59** | **-7.45** |
|  | **birth** | **-9.49** | **-14.19** | **-4.52** | **-9.93** | **-15.26** | **-4.28** |
|  | **2 years** | **-10.53** | **-16.02** | **-4.69** | **-11.07** | **-16.39** | **-5.41** |
| **O_3_*^b^*** | **3rd trimester** | 1.41 | -4.18 | 7.33 | 4.63 | -2.20 | 11.93 |
|  | **birth** | -2.90 | -7.26 | 1.66 | -1.19 | -6.32 | 4.23 |
|  | **2 years** | -0.63 | -6.51 | 5.63 | -0.69 | -6.43 | 5.40 |
| Model 1 includes infant age, two spline terms, interactions between air pollutants and age and spline terms, and O_3_*^a^* or NO_2_*^b^* | | | | | | | |
| Model 2 further adjusted for maternal education level, maternal race, pre-pregnancy BMI, hypertensive disorders, diabetic disorders, recruitment site, maternal age, parity, breastfeeding duration, postnatal air pollution, and ambient temperature | | | | | | | |

| **Table S5.1.** Percent Difference in growth Comparing Change in Pregnancy-averaged Levels of Air Pollutants from 10^th^ to 90^th^ Percentile (Single-Pollutant Models) for Males | | | | | | | | | |
| --- | --- | --- | --- | --- | --- | --- | --- | --- | --- |
|  |  |  |  |  |  |  |  |  |  |
|  |  | **Model 1** | | |  | **Model 2** | | |  |
|  |  | **Percent Difference in Growth** | **95% CI** | | **P value** | **Percent Difference in Growth** | **95% CI** | | **P value** |
| **PM_2.5_** | **3rd trimester to 2 years** | 1.10 | 0.99 | 1.22 | 0.07 | 1.08 | 0.97 | 1.20 | 0.18 |
|  | **3rd trimester to 3 months** | **1.55** | **1.07** | **2.24** | **0.02** | 1.41 | 0.91 | 2.21 | 0.13 |
|  | **3 months to 6 months** | 1.34 | 0.84 | 2.14 | 0.23 | 1.37 | 0.93 | 2.03 | 0.11 |
|  | **6 months to 2 years** | 0.95 | 0.83 | 1.09 | 0.47 | 0.94 | 0.84 | 1.06 | 0.31 |
| **PM_10_** | **3rd trimester to 2 years** | 1.02 | 0.91 | 1.15 | 0.69 | 0.94 | 0.83 | 1.05 | 0.27 |
|  | **3rd trimester to 3 months** | 1.30 | 0.86 | 1.95 | 0.21 | 0.83 | 0.52 | 1.32 | 0.43 |
|  | **3 months to 6 months** | 1.15 | 0.69 | 1.91 | 0.60 | 1.34 | 0.88 | 2.03 | 0.17 |
|  | **6 months to 2 years** | 0.93 | 0.78 | 1.10 | 0.39 | 0.92 | 0.80 | 1.05 | 0.22 |
| **NO_2_** | **3rd trimester to 2 years** | 1.05 | 0.95 | 1.17 | 0.35 | 1.10 | 0.99 | 1.22 | 0.09 |
|  | **3rd trimester to 3 months** | **1.69** | **1.14** | **2.51** | **0.01** | **1.90** | **1.21** | **2.98** | **0.01** |
|  | **3 months to 6 months** | 1.03 | 0.64 | 1.67 | 0.90 | 1.06 | 0.71 | 1.57 | 0.78 |
|  | **6 months to 2 years** | 0.90 | 0.78 | 1.04 | 0.17 | 0.92 | 0.82 | 1.04 | 0.17 |
| **O_3_** | **3rd trimester to 2 years** | 0.96 | 0.87 | 1.06 | 0.39 | **0.88** | **0.80** | **0.98** | **0.02** |
|  | **3rd trimester to 3 months** | 0.70 | 0.49 | 1.01 | 0.05 | **0.48** | **0.31** | **0.73** | **0.00** |
|  | **3 months to 6 months** | 1.11 | 0.70 | 1.74 | 0.67 | 1.25 | 0.85 | 1.83 | 0.25 |
|  | **6 months to 2 years** | 1.04 | 0.90 | 1.19 | 0.63 | 1.02 | 0.91 | 1.15 | 0.68 |
| CI: confidence interval; | | | | | | | | |  |
| Model 1 includes infant age, two spline terms, and interactions between air pollutants and age and spline terms | | | | | | | | |  |
| Model 2 further adjusted for maternal education level, maternal race, pre-pregnancy BMI, hypertensive disorders, diabetic disorders, recruitment site, maternal age, parity, breastfeeding duration, postnatal air pollutant (same as the exposure), and ambient temperature | | | | | | | | |  |

| **Table S5.2.** Percent Difference in growth Comparing Change in Pregnancy-averaged Levels of Air Pollutants from 10^th^ to 90^th^ Percentile (Single-Pollutant Models) for Females | | | | | | | | | |
| --- | --- | --- | --- | --- | --- | --- | --- | --- | --- |
|  |  |  |  |  |  |  |  |  |  |
|  |  | **Model 1** | | |  | **Model 2** | | |  |
|  |  | **Percent Difference in Growth** | **95% CI** | | **P value** | **Percent Difference in Growth** | **95% CI** | | **P value** |
| **PM_2.5_** | **3rd trimester to 2 years** | 1.04 | 0.94 | 1.15 | 0.50 | 1.02 | 0.93 | 1.11 | 0.75 |
|  | **3rd trimester to 3 months** | **1.40** | **0.99** | **1.97** | **0.05** | 1.35 | 0.95 | 1.91 | 0.09 |
|  | **3 months to 6 months** | 1.12 | 0.72 | 1.74 | 0.63 | 1.04 | 0.74 | 1.46 | 0.82 |
|  | **6 months to 2 years** | 0.92 | 0.80 | 1.07 | 0.29 | 0.92 | 0.82 | 1.03 | 0.16 |
| **PM_10_** | **3rd trimester to 2 years** | 0.97 | 0.86 | 1.09 | 0.55 | 0.97 | 0.87 | 1.07 | 0.51 |
|  | **3rd trimester to 3 months** | 0.99 | 0.70 | 1.42 | 0.97 | 1.16 | 0.79 | 1.70 | 0.44 |
|  | **3 months to 6 months** | 1.25 | 0.77 | 2.03 | 0.36 | 1.00 | 0.69 | 1.46 | 1.00 |
|  | **6 months to 2 years** | 0.92 | 0.77 | 1.09 | 0.33 | 0.90 | 0.78 | 1.04 | 0.15 |
| **NO_2_** | **3rd trimester to 2 years** | 1.03 | 0.94 | 1.14 | 0.53 | 0.98 | 0.90 | 1.08 | 0.70 |
|  | **3rd trimester to 3 months** | **1.56** | **1.11** | **2.21** | **0.01** | 1.32 | 0.91 | 1.91 | 0.14 |
|  | **3 months to 6 months** | 1.03 | 0.66 | 1.60 | 0.91 | 1.00 | 0.71 | 1.42 | 0.99 |
|  | **6 months to 2 years** | 0.90 | 0.78 | 1.03 | 0.13 | **0.89** | **0.79** | **0.99** | **0.03** |
| **O_3_** | **3rd trimester to 2 years** | 1.01 | 0.92 | 1.10 | 0.89 | 1.01 | 0.93 | 1.10 | 0.81 |
|  | **3rd trimester to 3 months** | **0.65** | **0.48** | **0.89** | **0.01** | **0.70** | **0.50** | **0.98** | **0.04** |
|  | **3 months to 6 months** | 1.03 | 0.68 | 1.56 | 0.88 | 1.00 | 0.72 | 1.38 | 0.98 |
|  | **6 months to 2 years** | **1.16** | **1.01** | **1.32** | **0.03** | **1.14** | **1.03** | **1.27** | **0.01** |

| **Table S5.3.** Percent Difference in Mean Weight Comparing Change in Pregnancy-averaged Levels of Air Pollutants from 10^th^ to 90^th^ Percentile (Single-Pollutant Models) for Males | | | | | | | | | |
| --- | --- | --- | --- | --- | --- | --- | --- | --- | --- |
|  |  |  |  |  |  |  |  |  |  |
| **Air Pollutant** | **Age** | **Model 1** | | |  | **Model 2** | | |  |
|  |  | **Percent Difference in Weight** | **95% CI** | | **P value** | **Percent Difference in Weight** | **95% CI** | | **P value** |
| **PM_2.5_** | **3rd trimester** | **-8.46** | **-14.46** | **-2.04** | **0.01** | **-8.32** | **-15.23** | **-0.85** | **0.03** |
|  | **birth** | -3.84 | -8.34 | 0.88 | 0.11 | -4.56 | -8.98 | 0.08 | 0.05 |
|  | **2 years** | 0.68 | -6.74 | 8.69 | 0.86 | 0.23 | -6.38 | 7.31 | 0.95 |
| **PM_10_** | **3rd trimester** | **-7.68** | **-14.11** | **-0.78** | **0.03** | 1.44 | -6.84 | 10.46 | 0.74 |
|  | **birth** | **-5.04** | **-9.57** | **-0.29** | **0.04** | -0.87 | -6.14 | 4.70 | 0.75 |
|  | **2 years** | -5.52 | -13.97 | 3.75 | 0.23 | -4.50 | -12.37 | 4.07 | 0.29 |
| **NO_2_** | **3rd trimester** | **-8.84** | **-15.09** | **-2.14** | **0.01** | **-12.40** | **-19.05** | **-5.20** | **0.00** |
|  | **birth** | -3.16 | -7.78 | 1.71 | 0.20 | **-5.36** | **-9.71** | **-0.79** | **0.02** |
|  | **2 years** | -3.67 | -11.01 | 4.26 | 0.35 | -3.73 | -10.20 | 3.21 | 0.28 |
| **O_3_** | **3rd trimester** | 2.02 | -4.25 | 8.71 | 0.54 | **12.34** | **4.30** | **20.99** | **0.00** |
|  | **birth** | -2.07 | -6.24 | 2.30 | 0.35 | 2.90 | -1.59 | 7.60 | 0.21 |
|  | **2 years** | -2.63 | -9.84 | 5.15 | 0.50 | -1.18 | -7.90 | 6.04 | 0.74 |
| CI: confidence interval; | | | | | | | | |  |
| Model 1 includes infant age, two spline terms, and interactions between air pollutants and age and spline terms | | | | | | | | |  |
| Model 2 further adjusted for maternal education level, maternal race, pre-pregnancy BMI, hypertensive disorders, diabetic disorders, recruitment site, maternal age, parity, breastfeeding duration, postnatal air pollution, and ambient temperature | | | | | | | | |  |

| **Table S5.4.** Percent Difference in Mean Weight Comparing Change in Pregnancy-averaged Levels of Air Pollutants from 10^th^ to 90^th^ Percentile (Single-Pollutant Models) for Females | | | | | | | | | |
| --- | --- | --- | --- | --- | --- | --- | --- | --- | --- |
|  |  |  |  |  |  |  |  |  |  |
| **Air Pollutant** | **Age** | **Model 1** | | |  | **Model 2** | | |  |
|  |  | **Percent Difference in Weight** | **95% CI** | | **P value** | **Percent Difference in Weight** | **95% CI** | | **P value** |
| **PM_2.5_** | **3rd trimester** | **-10.81** | **-16.29** | **-4.97** | **0.00** | **-7.75** | **-13.59** | **-1.51** | **0.02** |
|  | **birth** | **-7.72** | **-11.80** | **-3.45** | **0.00** | **-4.81** | **-8.82** | **-0.61** | **0.03** |
|  | **2 years** | **-8.01** | **-14.87** | **-0.60** | **0.03** | **-7.20** | **-13.07** | **-0.93** | **0.03** |
| **PM_10_** | **3rd trimester** | -5.26 | -11.24 | 1.13 | 0.10 | -6.00 | -12.70 | 1.21 | 0.10 |
|  | **birth** | **-5.99** | **-10.30** | **-1.47** | **0.01** | -4.61 | -9.26 | 0.28 | 0.06 |
|  | **2 years** | **-9.34** | **-17.70** | **-0.14** | **0.05** | **-10.76** | **-17.89** | **-3.01** | **0.01** |
| **NO_2_** | **3rd trimester** | **-12.20** | **-17.64** | **-6.40** | **0.00** | **-9.22** | **-15.31** | **-2.70** | **0.01** |
|  | **birth** | **-8.28** | **-12.44** | **-3.91** | **0.00** | **-6.97** | **-11.07** | **-2.68** | **0.00** |
|  | **2 years** | **-10.13** | **-16.37** | **-3.42** | **0.00** | **-11.73** | **-17.00** | **-6.12** | **0.00** |
| **O_3_** | **3rd trimester** | **9.04** | **3.01** | **15.42** | **0.00** | **9.41** | **2.74** | **16.52** | **0.01** |
|  | **birth** | 4.16 | -0.02 | 8.52 | 0.05 | **5.62** | **1.37** | **10.04** | **0.01** |
|  | **2 years** | **9.97** | **2.51** | **17.96** | **0.01** | **10.49** | **4.11** | **17.26** | **0.00** |
| CI: confidence interval; | | | | | | | | |  |
| Model 1 includes infant age, two spline terms, and interactions between air pollutants and age and spline terms | | | | | | | | |  |
| Model 2 further adjusted for maternal education level, maternal race, pre-pregnancy BMI, hypertensive disorders, diabetic disorders, recruitment site, maternal age, parity, breastfeeding duration, postnatal air pollution, and ambient temperature | | | | | | | | |  |

| **Table S6.** Percent Difference in Growth Comparing Change in Pregnancy-averaged Levels of Air Pollutants from 10^th^ to 90^th^ Percentile (exclude subjects with pre-term birth) | | | | | | | | | |
| --- | --- | --- | --- | --- | --- | --- | --- | --- | --- |
|  |  |  |  |  |  |  |  |  |  |
|  |  | **Model 1** | | |  | **Model 2** | | |  |
|  |  | **Percent Difference in Growth** | **95% CI** | | **P value** | **Percent Difference in Growth** | **95% CI** | | **P value** |
| **PM_2.5_** | **3rd trimester to 2 years** | 1.05 | 0.99 | 1.12 | 0.14 | 1.02 | 0.96 | 1.09 | 0.52 |
|  | **3rd trimester to 3 months** | **1.62** | **1.28** | **2.03** | **0.00** | **1.38** | **1.05** | **1.82** | **0.02** |
|  | **3 months to 6 months** | 1.00 | 0.75 | 1.35 | 0.98 | 1.02 | 0.80 | 1.31 | 0.86 |
|  | **6 months to 2 years** | 0.91 | 0.83 | 1.00 | 0.06 | **0.92** | **0.86** | **1.00** | **0.04** |
| **PM_10_** | **3rd trimester to 2 years** | 0.97 | 0.90 | 1.05 | 0.41 | 0.93 | 0.86 | 1.01 | 0.07 |
|  | **3rd trimester to 3 months** | 1.22 | 0.95 | 1.56 | 0.13 | 1.02 | 0.75 | 1.37 | 0.91 |
|  | **3 months to 6 months** | 0.98 | 0.70 | 1.36 | 0.90 | 0.96 | 0.73 | 1.27 | 0.77 |
|  | **6 months to 2 years** | 0.90 | 0.80 | 1.01 | 0.06 | **0.90** | **0.82** | **0.99** | **0.03** |
| **NO_2_** | **3rd trimester to 2 years** | 0.98 | 0.92 | 1.05 | 0.53 | 0.99 | 0.93 | 1.06 | 0.82 |
|  | **3rd trimester to 3 months** | **1.50** | **1.18** | **1.90** | **0.00** | **1.51** | **1.14** | **2.00** | **0.00** |
|  | **3 months to 6 months** | 0.97 | 0.71 | 1.31 | 0.82 | 0.95 | 0.74 | 1.23 | 0.71 |
|  | **6 months to 2 years** | **0.85** | **0.77** | **0.94** | **0.00** | **0.87** | **0.80** | **0.94** | **0.00** |
| **O_3_** | **3rd trimester to 2 years** | 1.02 | 0.96 | 1.09 | 0.45 | 0.99 | 0.93 | 1.06 | 0.86 |
|  | **3rd trimester to 3 months** | **0.69** | **0.55** | **0.86** | **0.00** | **0.64** | **0.49** | **0.83** | **0.00** |
|  | **3 months to 6 months** | 1.06 | 0.79 | 1.41 | 0.70 | 1.07 | 0.84 | 1.36 | 0.61 |
|  | **6 months to 2 years** | **1.16** | **1.06** | **1.28** | **0.00** | **1.14** | **1.05** | **1.23** | **0.00** |
| CI: confidence interval; | | | | | | | | |  |
| Model 1 includes infant age, two spline terms, and interactions between air pollutants and age and spline terms | | | | | | | | |  |
| Model 2 further adjusted for maternal education level, maternal race, pre-pregnancy BMI, hypertensive disorders, diabetic disorders, recruitment site, maternal age, parity, breastfeeding duration, postnatal air pollutant (same as the exposure), and ambient temperature | | | | | | | | |  |

| **Table S7.** Percent Difference in Mean Weight Comparing Change in Pregnancy-averaged Levels of Air Pollutants from 10^th^ to 90^th^ Percentile (exclude subjects with pre-term birth) | | | | | | | | | |
| --- | --- | --- | --- | --- | --- | --- | --- | --- | --- |
|  |  |  |  |  |  |  |  |  |  |
| **Air Pollutant** | **Age** | **Model 1** | | |  | **Model 2** | | |  |
|  |  | **Percent Difference in Weight** | **95% CI** | | **P value** | **Percent Difference in Weight** | **95% CI** | | **P value** |
| **PM_2.5_** | **3rd trimester** | **-8.83** | **-12.92** | **-4.56** | **0.00** | -5.56 | -10.91 | 0.12 | 0.05 |
|  | **birth** | **-3.84** | **-6.91** | **-0.67** | **0.02** | -2.09 | -5.85 | 1.82 | 0.29 |
|  | **2 years** | -4.37 | -9.25 | 0.77 | 0.09 | -3.49 | -7.91 | 1.14 | 0.14 |
| **PM_10_** | **3rd trimester** | **-5.98** | **-10.47** | **-1.27** | **0.01** | -2.40 | -8.54 | 4.16 | 0.46 |
|  | **birth** | **-3.92** | **-7.13** | **-0.61** | **0.02** | -2.21 | -6.48 | 2.26 | 0.33 |
|  | **2 years** | **-8.99** | **-14.86** | **-2.71** | **0.01** | **-9.14** | **-14.49** | **-3.45** | **0.00** |
| **NO_2_** | **3rd trimester** | **-6.92** | **-11.19** | **-2.43** | **0.00** | **-7.63** | **-12.96** | **-1.97** | **0.01** |
|  | **birth** | **-2.63** | **-5.80** | **0.65** | **0.11** | -3.32 | -7.06 | 0.57 | 0.09 |
|  | **2 years** | **-8.82** | **-13.61** | **-3.75** | **0.00** | **-8.34** | **-12.61** | **-3.87** | **0.00** |
| **O_3_** | **3rd trimester** | **3.96** | **-0.33** | **8.43** | **0.07** | **6.83** | **1.18** | **12.81** | **0.02** |
|  | **birth** | -0.24 | -3.13 | 2.73 | 0.87 | 1.69 | -1.91 | 5.43 | 0.36 |
|  | **2 years** | 6.48 | 1.05 | 12.20 | **0.02** | **6.21** | **1.39** | **11.25** | **0.01** |
| CI: confidence interval; | | | | | | | | |  |
| Model 1 includes infant age, two spline terms, and interactions between air pollutants and age and spline terms | | | | | | | | |  |
| Model 2 further adjusted for maternal education level, maternal race, pre-pregnancy BMI, hypertensive disorders, diabetic disorders, recruitment site, maternal age, parity, breastfeeding duration, postnatal air pollution, and ambient temperature | | | | | | | | |  |

**
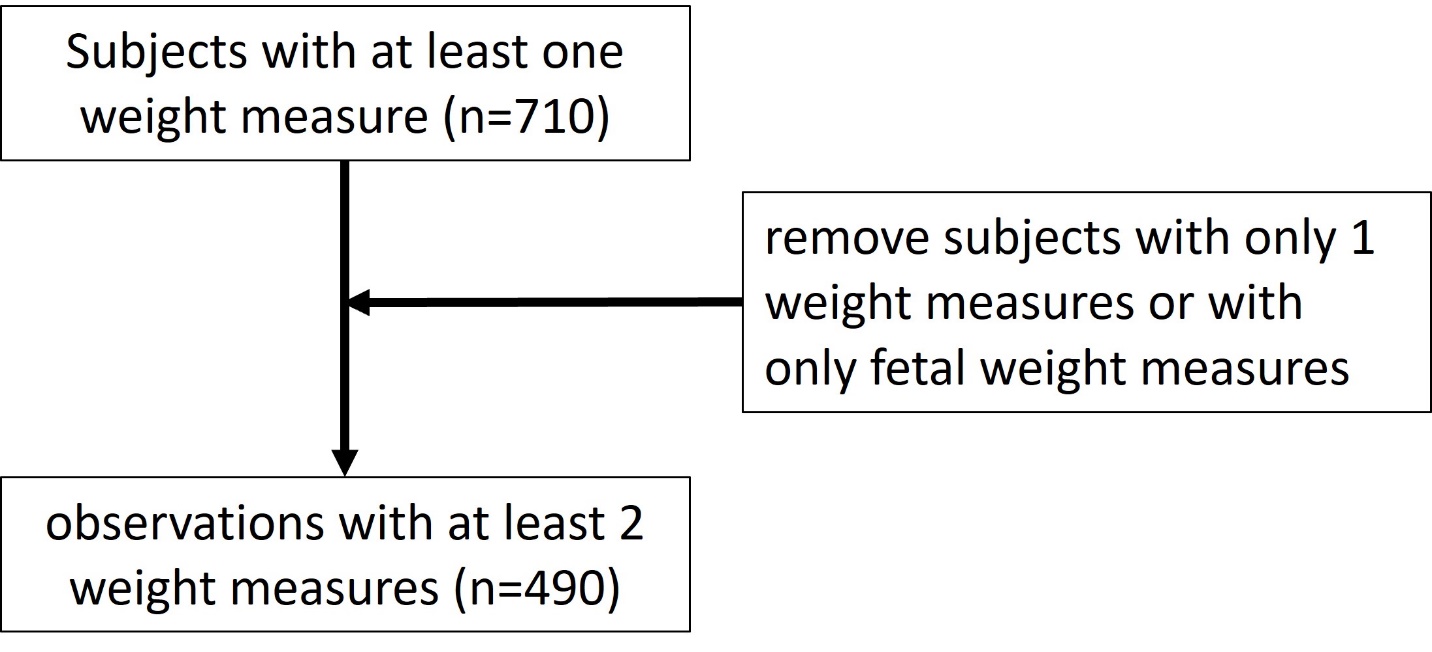
**

**Figure S1.** Conceptual Flow Chart

**
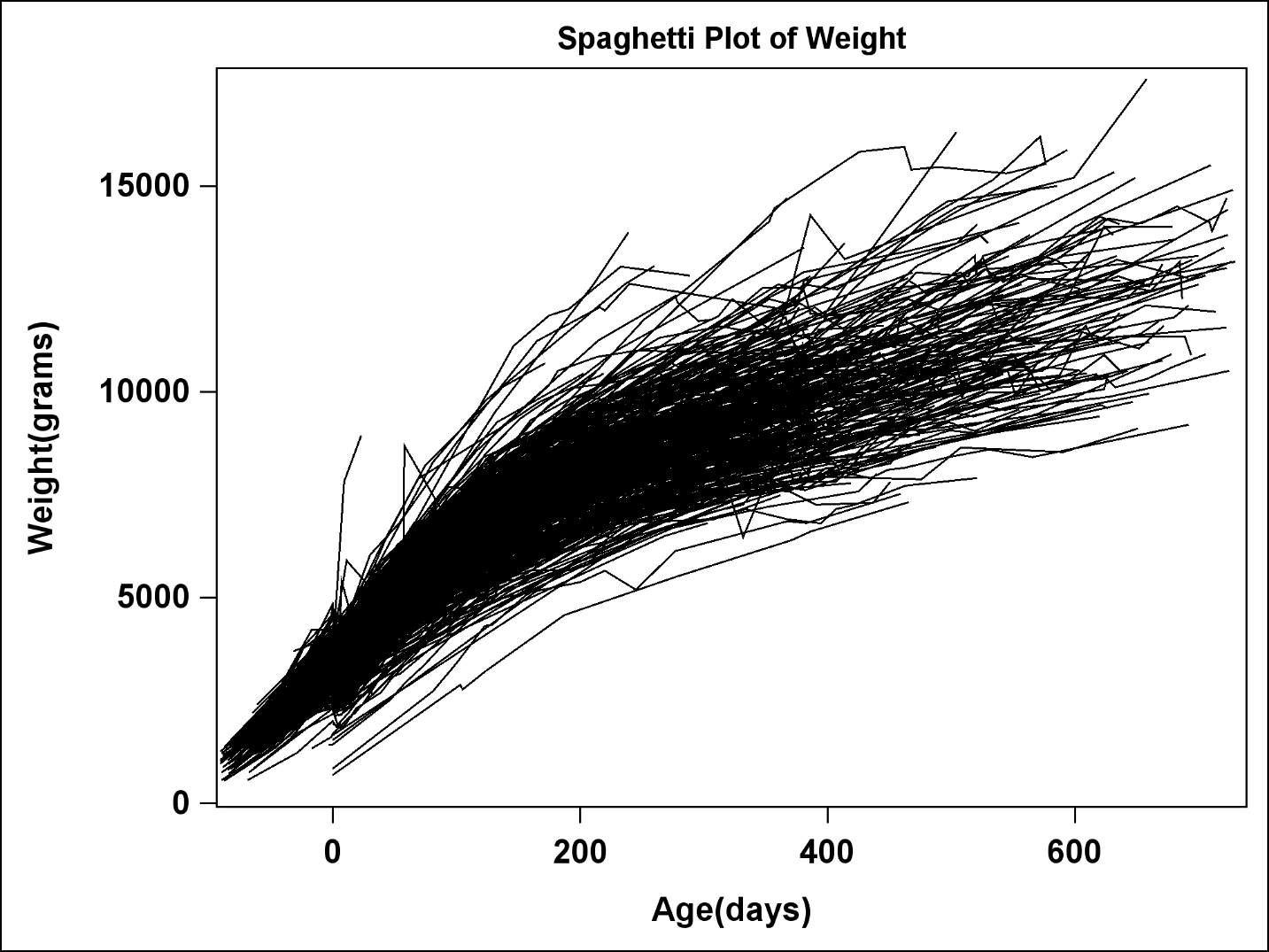
**

**Figure S2.** Spaghetti Plot of Weight


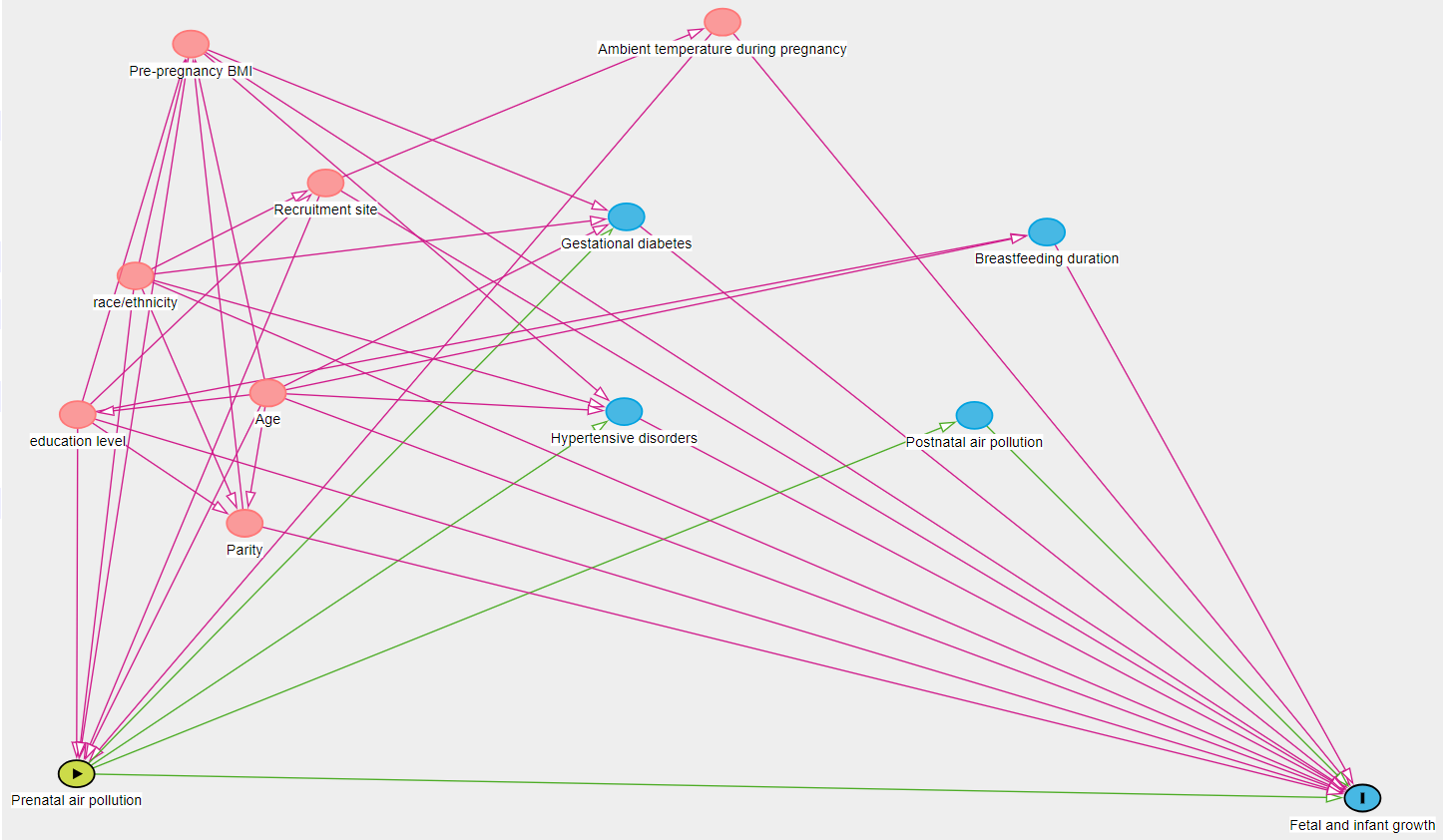


**Figure S3.** Directed Acyclic Graph (DAG) Of the Associations Between Prenatal Air Pollution and Child Weight Trajectory

**
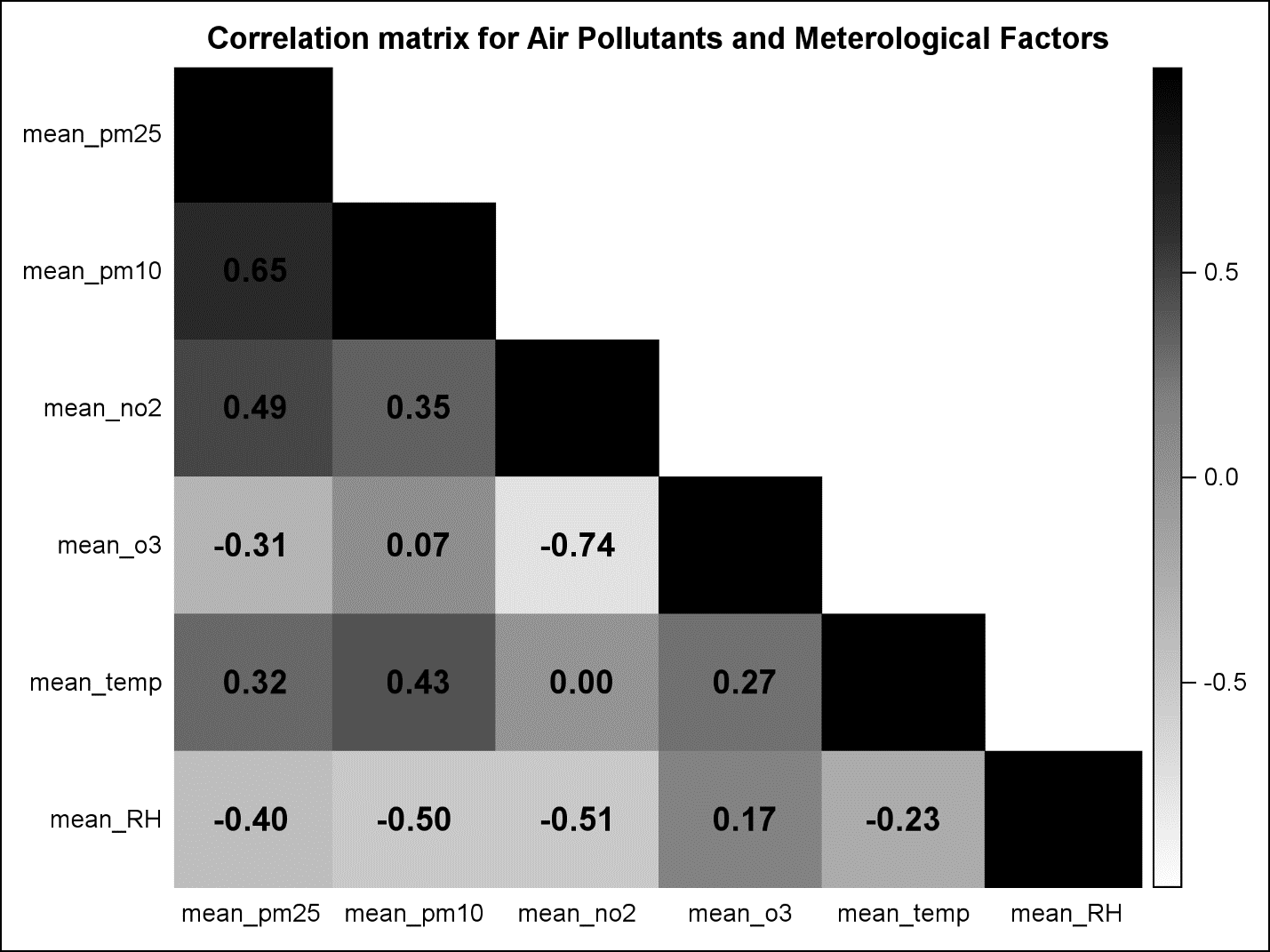
**

**Figure S4.** Correlation Matrix for Air Pollutants and Meteorological factors
